# Characterization of Fructan Metabolism During Jerusalem Artichoke (*Helianthus tuberosus* L.) Germination

**DOI:** 10.3389/fpls.2018.01384

**Published:** 2018-09-19

**Authors:** Jiao Jiao, Ji Wang, Mengjia Zhou, Xuyang Ren, Wenyue Zhan, Zongjiu Sun, Haiyan Zhao, Yao Yang, Mingxiang Liang, Wim Van den Ende

**Affiliations:** ^1^College of Resources and Environmental Sciences, Nanjing Agricultural University, Nanjing, China; ^2^Jiangsu Key Lab of Marine Biology, Nanjing, China; ^3^College of Grassland and Environmental Sciences, Xinjiang Agricultural University, Ürümqi, China; ^4^Department of Food Science and Technology, Jinling College, Nanjing Normal University, Nanjing, China; ^5^Laboratory of Molecular Plant Biology, KU Leuven, Leuven, Belgium

**Keywords:** enzyme activity, gene expression, jerusalem artichoke, tuber sprouting, sugar

## Abstract

The inulin-type fructans in Jerusalem artichoke (*Helianthus tuberosus* L.) tubers exhibit different degrees of polymerization and are critical for germination. We aimed to characterize the sugar metabolism dynamics in the tubers without bud eyes or shoots (T) and BE/S of indoor- and field-grown Jerusalem artichokes during germination. *Ht1-FEH II* and *Ht1-FEH III* (1-fructan exohydrolases II and III, inulin-degrading enzymes) expression increased 5 days after planting indoors, whereas *Ht1-FEH II* expression increased 72 days after planting in the field in T and BE/S. *Ht1-SST* (sucrose:sucrose 1-fructosyl transferase, inulin synthesis initiator), and *Ht1-FFT* (fructan:fructan 1-fructosyl transferase, inulin elongator) expression generally decreased in indoor-grown T. The enzyme activities of 1-FEH and 1-FFT were unchanged during germination in both indoor- and field-grown T and BE/S, whereas 1-SST activity decreased in indoor-grown T, while 1-FEH and 1-FFT activities increased as a function of germination time in BE/S of both indoor- and field-grown tubers. The total soluble sugar content gradually decreased in T after germination indoors or in the field, while at the end of germination, the sucrose and fructan contents decreased, and fructose content increased in the field. The enzyme activities of soluble vacuolar (VI) or neutral invertase (NI) did not change significantly, except at the late germination stage. Sucrose synthase (SS) and sucrose-phosphate synthase (SPS) activities were not significantly changed in T and BE/S in indoor-grown artichokes, while SS activity gradually increased, and SPS activity gradually decreased in field-grown artichokes, alongside sucrose degradation. Compared to T, BE/S generally had higher enzyme activities of 1-FEH and 1-FFT, promoting inulin hydrolysis. This work shows that the process of tuber germination is similar indoors and in the field, and germination studies can therefore be conducted in either environment.

## Introduction

Jerusalem artichoke (*Helianthus tuberosus* L.) is a herbaceous perennial plant that grows to around 2 m in height. Due to its high biomass yield and tolerance to abiotic stress, Jerusalem artichoke is extensively cultivated on marginal lands for vegetable production or for wind prevention and sand fixation (Conde et al., 1991; Puangbut et al., 2015). Jerusalem artichoke accumulates inulin, a type of fructan, in the tuber, and this accounts for approximately 85% of the tuber’s dry weight (Marx et al., 1997). Jerusalem artichoke inulin consists of linear β (2, 1)-linked fructofuranosyl units terminated by Glc. The DP, fructofuranosyl units) of inulin varies throughout the growing season, with inulin in mature tubers containing 3 to 35 fructofuranosyl units but fructan DP 2–10 constituting the majority (Luo et al., 2018). Inulin is commonly included in bread and yogurt products as a prebiotic, as it stimulates the growth of beneficial bacteria in the colon (Ritsema and Smeekens, 2003; Drabinska et al., 2016).

Sucrose:sucrose 1-fructosyl transferase (1-SST) and fructan:fructan 1-fructosyl transferase (1-FFT) catalyze the synthesis of inulin from Suc in Jerusalem artichoke tubers (Van der Meer et al., 1998). By contrast, 1-FEH catalyzes the degradation of inulin, hydrolyzing it to Fru and Suc (Marx et al., 1997). In Jerusalem artichoke, only one 1-SST and one 1-FFT have been reported, but there are three 1-FEH members (Ht1-FEH I, Ht1-FEH II, and Ht1-FEH III) (Xu et al., 2015; Zhan et al., 2018).

Invertase irreversibly degrades Suc into Glc and Fru. Here, we consider two types of soluble INVs, vacuolar INV (VI), and cytosolic alkaline/neutral INV (NI) with an acidic and neutral to basic pH optimum, respectively (Sturm, 1999; Wan et al., 2018). Plant 1-SSTs, 1-FFTs, 1-FEHs, and VIs belong to the plant glycoside hydrolase family 32 (GH32) and are thought to have evolved from ancestral INVs due to their high levels of sequence similarity (Lammens et al., 2009; Van den Ende et al., 2009). The preferential substrate specificity within GH32 members can be modulated by several amino acid mutations (Verhaest et al., 2007; Schroeven et al., 2008; Lasseur et al., 2009). NIs belong to another glycoside hydrolase family (GH100).

Suc is synthesized by cytoplasmic SPS and SPP in source leaves. The reaction catalyzed by SPS is considered to be the limiting step in Suc synthesis (Winter and Huber, 2000). Suc is transported from its source (e.g., leaves) through the phloem to sink tissues (e.g., tubers), and is then reversibly hydrolyzed by SS or INV into (UDP-)Glc and Fru in sink tissues (Ruan, 2014).

Tubers expand and reach maturity by accumulating Suc (Soja et al., 1989). After dormancy, the tubers start to germinate. The potato (*Solanum tuberosum*) tuber undergoes a sink–source transition during germination (Viola et al., 2007). We previously showed that fructan metabolism enzymes (such as 1-FEHs) play critical roles in inulin hydrolysis after tuber germination (Xu et al., 2015). To improve our understanding of the tuber germination process, we characterized sugar metabolism during germination, focusing both on fructan and Suc metabolism and on changes in sugar profiles.

## Materials and Methods

### Plant Materials and Growth Conditions

Jerusalem artichoke variety Nanyu No. 1 was used in this study. All tubers were grown in the field at an experimental station at Nanjing Agricultural University, Dafeng, Jiangsu Province. Similar sized tubers were rinsed with tap water, distilled water, and 1 g/L sodium stearate and then left to dry in a cool, ventilated place. Tubers were divided in two groups: one group was planted again in another field while another group was planted indoors and germination was compared between the two groups.

For indoor germination experiments, tubers were covered in quartz sand and kept moist in a 20°C incubator until germination without light. At least four tubers with three biological replicates were sampled and peeled at 0, 2, 4, and 5 DAP. For the field germination experiments, tubers were planted in soil at the Pailou experimental station of Nanjing Agricultural University from January 23 to April 12, 2017. At least seven tubers were grown, with three replicates, until 14, 28, 40, 45, 61, and 72 DAP. Tubers were sampled, cleaned, peeled, rapidly ground in liquid nitrogen, and stored at -80°C until use. Bud eyes on the tuber are prominent (around 0.5 cm in height) and easily distinguishable from the tubers. As a consequence, bud eyes and shoots developing from them (abbreviated as BE/S from this point on) could be easily separated from the tubers (abbreviated as T from this point on; T does not comprise BE/S material), by cutting them along the tuber’s surface. For indoor germination, T and BE/S were sampled at 0, 2, 4, and 5 DAP. For field germination, T and BE/S were sampled at 0, 14, 28, 40, 45, 61, and 72 DAP. However, in the latter case each BE/S was further separated in 1 cm segments (BE/S0: proximal part, 0–1 cm; BE/S1: intermediate part, 1–2 cm; and BE/S2: distal part, >2 cm) for gene expression analysis. For all enzyme activity and sugar measurements, full T and BE/S were used.

### Total RNA Isolation and cDNA Synthesis

Total RNA was extracted from T and BE/S materials using a TRIzol Reagent Total RNA Extraction Kit (CW0580S) according to the manufacturer’s instructions. cDNA was obtained by a two-step method (TaKaRa; D6110A).

### Gene Expression Analysis by Quantitative PCR (qPCR)

Quantitative PCR (qPCR) assays and data analysis were performed as previously described (Xu et al., 2015). The levels of *Ht1-FEH I, Ht1-FEH II, Ht1-FEH III, Ht1-FFT*, and *Ht1-SST* transcripts were quantified by qPCR using a 7,500 Real Time PCR System (Applied Biosystems) and SYBR Premix Ex Taq (Takara, DRR041A) according to the manufacturer’s instructions. Data were processed using the 2^-ΔΔCT^ method. The expression level of *Ht1-FEH I, Ht1-FEH II, Ht1-FEH III, Ht1-FFT*, and *Ht1-SST* relative to the Jerusalem artichoke housekeeping gene *Actin* was calculated and compared to the expression levels in 0-day samples, which were set to 1.0. Three biological and three technical repeats were performed for each sample.

### Protein Extraction and Enzyme Activity Measurements

#### Soluble Protein Extracts Containing 1-FEH, 1-FFT and 1-SST Enzymes, and Measurements of Their Activities

Soluble protein extracts containing 1-FEH, 1-FFT and 1-SST were extracted from T and BE/S according to previously described methods (Joudi et al., 2012; Vandoorne et al., 2012).

The protein concentration was measured using a TaKaRa Bradford Protein Assay Kit (Code No. T9310A) with BSA as a standard, following the manufacturer’s protocol. The activity of 1-FEH and 1-FFT were assayed by incubating 100 μl soluble protein extract with 900 μL 5 mM 1-K at 30°C (1-FEH) or on ice (1-FFT) for 4 h. The activity of 1-SST was assayed by adding 900 μL 100 mM Suc to 100 μL soluble protein extract, followed by incubation at 30°C for 4 h. All reactions were stopped by incubating the samples at 95°C for 5 min and then filtering them through a 0.45-μm membrane after cooling to room temperature. The reaction products (1-FEH: Fru; 1-FFT: N; 1-SST: 1-K) were quantified by high-performance liquid chromatography (HPLC) as described (Xu et al., 2015).

#### Soluble Protein Extracts Containing VI, NI, SPS and SS Enzymes, and Measurements of Their Activities

The activities of VI and NI were measured as described (Dai et al., 2016) with modifications. The VI activity was assayed by incubating 50 μL of crude enzyme with 50 μL of reaction buffer (0.1 M acetic acid buffer pH 5.5 and 30 mM Suc) at 30°C for 1 h. Then, 1 mL of 27.6 mM 3, 5-dinitrosalicylic acid was added and the sample was incubated in a water bath at 100°C for 5 min. The mixture was brought to 2 mL with distilled water after cooling to room temperature and assayed at a wavelength of 540 nm. As a control, 50 μL of crude enzyme, inactivated by boiling for 3 min, was used. The rate of reducing sugar formation, a measure of INV activity, was calculated based on the difference between the boiled crude enzyme and crude enzyme preparations. The assay for NI activity was similar to that of VI except that the reaction was performed in phosphate buffer (pH 7.5). All reactions were stopped by incubating the samples at 95°C for 5 min.

Sucrose 6-phosphate synthase (SPS) activity was assayed by incubating 30 μL of crude enzyme with 40 μL of reaction buffer (15 mM MgCl_2_, 5 mM fructose-6-phosphate, 15 mM glucose-6-phosphate, and 10 mM UDP-Glc) at 30°C for 30 min. The reaction was stopped by adding 70 μL of 3 M KOH. Background was determined by adding the stopping agent before adding the enzyme. The reaction mixture for the SS assay was similar to that for the SPS assay, but it contained 60 mM Fru instead of 5 mM fructose-6-phosphate and was devoid of glucose-6-phosphate. Enzyme activities were determined using the phenol-sulfuricacidmethod.

### Total Soluble Sugar Measurements

The total soluble sugar content was measured using the anthrone method. Sugar was extracted from tubers and quantified according to our previously described method (Xu et al., 2015). Fru, Glc, and Suc standards were purchased from SIGMA and 1-K, N, and 1^F^-N were purchased from Wako Pure Chemical Industries, Ltd.

### Statistical Analysis

One-way ANOVA using the LSD method was conducted in SPSS 19.0 (SPSS Corp., Chicago, IL, United States) to evaluate significant differences among treatments. Figures were drawn using SigmaPlot 10 (Systat Software, Inc., Germany) software.

## Results

### Relative Expression of *Ht1-FEH I, Ht1-FEH II, Ht1-FEH III, Ht1-FFT*, and *Ht1-SST* During Tuber Germination

Tubers were germinated in two environments, indoors at a constant temperature of 20°C and in the field under regular production conditions (from January 23^rd^ to April 12th; the average temperature is listed in **Supplementary Table S1**). Photographs of germinating tubers are shown in **Supplementary Figure S1**. Generally, shoots were visible at 5 DAP for tubers grown indoors and at 40 DAP for those in the field.

**FIGURE 1:**
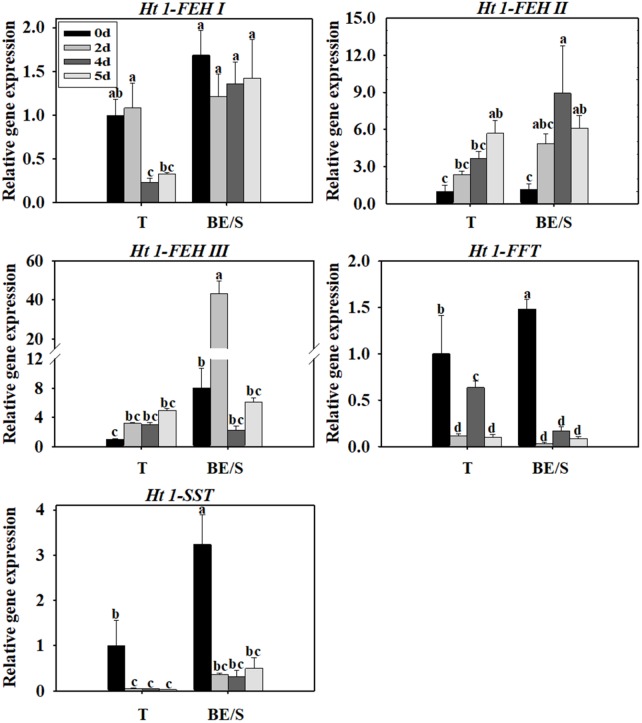
Relative expression of *Ht1-FEH I*, *Ht1-FEH II*, *Ht1-FEH III*, *Ht1-FFT*, and *Ht1-SST* in T and BE/S of Jerusalem artichoke grown indoors. Samples were collected at 0, 2, 4, and 5 days after planting (DAP). T, tuber without bud eyes and shoots; BE/S, bud eyes plus shoots. Values represent mean ± SE of three biological replicates. Different letters indicate significant differences compared to 0 DAP for each tissue.

**FIGURE 2:**
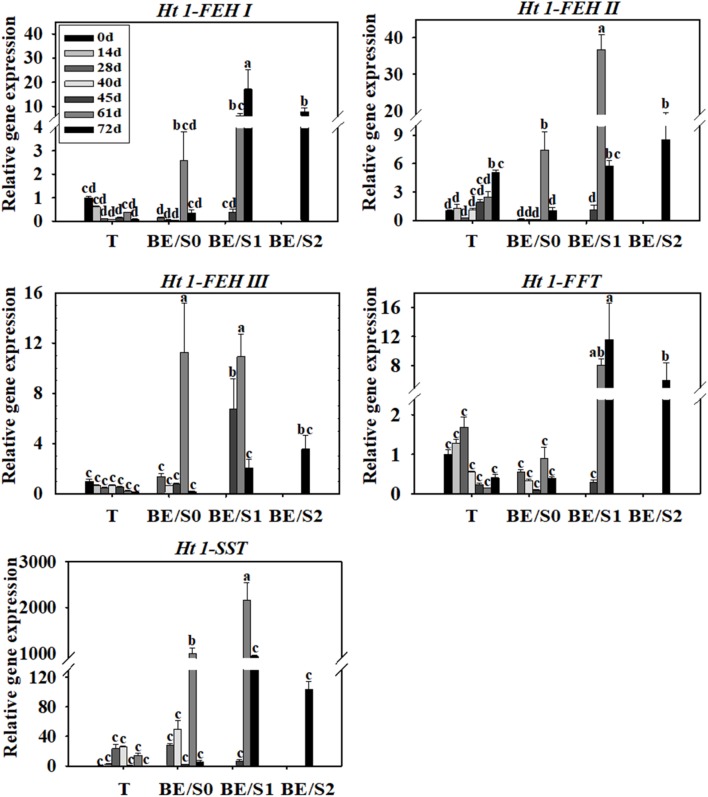
Relative expression of *Ht1-FEH I*, *Ht1-FEH II*, *Ht1-FEH III*, *Ht1-FFT*, and *Ht1-SST* in T and BE/S of Jerusalem artichoke grown in the field. Samples were collected at 0, 14, 28, 40, 45, 61, and 72 DAP. T, tuber without bud eyes and shoots; BE/S0, proximal part, 0–1 cm; BE/S1, intermediate part, 1–2 cm; and BE/S2: distal part, >2 cm. Values represent mean ± SE of three biological replicates. Different letters indicate significant differences compared to 0 DAP for each tissue.

Tubers of Jerusalem artichokes contain bud eyes (BE). Shoots (S) first emerge from these BE (**Supplementary Figure S1**). To determine the roles of genes related to inulin metabolism during tuber germination, we quantified their expression levels in T and BE/S. *Ht1-FEH I* expression decreased in T, while remaining stable in BE/S, up to 5 DAP in tubers grown indoors (**Figure 1**; Primers for qPCR are listed in **Supplementary Table S2**). *Ht1-FEH I* expression kept unchanged in field-grown T from 0 DAP to 72 DAP (**Figure 2**). *Ht1-FEH II* expression was generally induced in T and BE/S during sprouting, both under indoor and field conditions (**Figures 1**, **2**). In the field (**Figure 2**), *Ht1-FEH II* transcript levels were highest toward the end of the sampling period in BE/S1. *Ht1-FEH III* transcripts accumulated in indoor grown T, and were most abundant in BE/S at 2 DAP, but gradually kept unchanged in T grown in the field. The expression level of *Ht1-FEH III* was usually higher in BE/S than in T.

Considering inulin biosynthesis, *Ht1-SST* and *Ht1-FFT* transcript levels generally decreased in T and BE/S grown indoors at 20°C, but *Ht1-SST* transcript levels increased in field-grown T. Transcripts of *Ht1-FFT* peaked in BE/S1 at 72 DAP.

### Activities of Enzymes Involved in Inulin Metabolism During Tuber Germination

**FIGURE 3:**
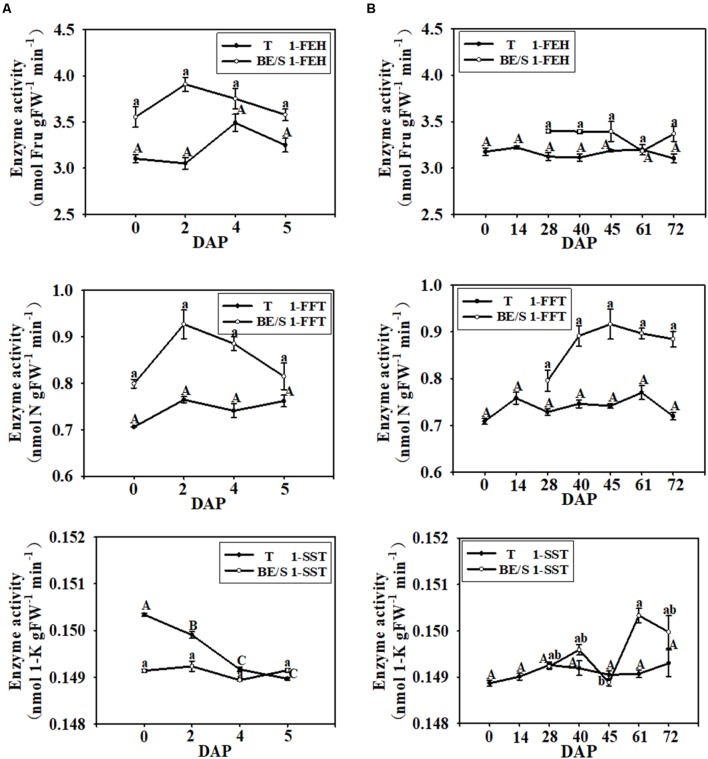
1-FEH, 1-FFT, and 1-SST activities in T and BE/S of Jerusalem artichokes grown indoors **(A)** and in the field **(B)**. For the indoor experiment, samples were collected at 0, 2, 4, and 5 DAP. For the field experiment, samples were collected at 0, 14, 28, 40, 45, 61, and 72 DAP. T, tuber without bud eyes and shoots; BE/S, bud eyes plus shoots; 1-K, 1-kestose; N, 1, 1-nystose; Fru, fructose. Values are means ± SE (*n* = 3). Different letters indicate significant differences compared to 0 DAP for each tissue.

Next, the activities of inulin metabolizing enzymes in Jerusalem artichokes were quantified indoors and in the field. In T cultured under both growth environments, the activities of 1-FEH and 1-FFT were relatively stable throughout germination, but the enzyme activities in BE/S were higher than in T, except at 61 DAP (**Figure 3**). In the indoor-grown Jerusalem artichokes, 1-SST activity was higher in T than in BE/S before 4 DAP, but at 5 DAP, 1-SST activity became lower in T than in BE/S. In the field, 1-SST activity was lower at 45 DAP.

### Sugar Contents During Tuber Germination

**FIGURE 4:**
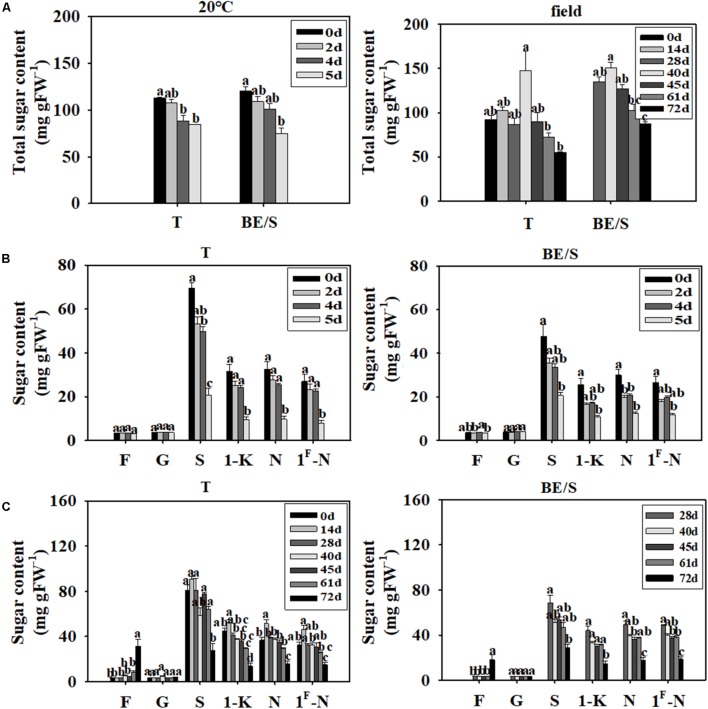
Total soluble sugar content in T and BE/S of indoor- and field-grown Jerusalem artichokes **(A)**. Sugar content in T and BE/S grown indoors **(B)** and in the field **(C)**. For the indoor experiment, samples were collected at 0, 2, 4, and 5 DAP. For the field experiment, samples were collected at 0, 14, 28, 40, 45, 61, and 72 DAP. T, tuber without bud eyes and shoots; BE/S, bud eyes plus shoots; F, fructose; G, glucose; S, sucrose; 1-K, 1-kestose; N, 1, 1-nystose; 1^F^-N, 1^F^-fructofuranosylnystose. Values are means ± SE (*n* = 3). Different letters indicate significant differences compared to 0 DAP for each tissue.

The total sugar content decreased throughout germination, both in T germinated indoors and in the field (**Figure 4A**). To further characterize the changes of various sugars during germination, we measured Fru, Glc, Suc, 1-K, N, and 1^F^-N levels by HPLC at different time points. Typical sugar profiles are listed in **Supplementary Figures S2**, **S3**. The T and BE/S from indoor germinating materials showed no notable differences in Fru and Glc content at 5 DAP as compared to 0 DAP (**Figure 4B**). However, the levels of Suc, 1-K, N, and 1^F^-N were 2-fold lower in both tissues at 5 DAP compared to 0 DAP.

The Glc content remained roughly constant throughout germination of field-grown tubers (**Figure 4C**). The Fru content was significantly higher, around 10-fold in tubers and 4-fold in shoots, at 72 DAP compared to 0 DAP. The contents of Suc, 1-K, N, and 1^F^-N in the field-grown tubers at 72 DAP were around 2-fold higher as compared to 28 DAP. Overall, sugar dynamics between T and BE/S samples were remarkably similar.

### The Activities of Enzymes Involved in Suc Metabolism During Tuber Germination

**FIGURE 5:**
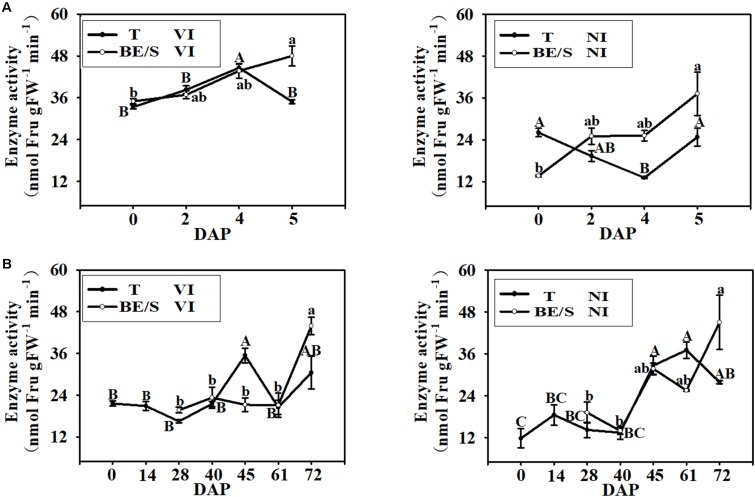
VI and NI enzyme activities in T and BE/S grown indoors **(A)** and in the field **(B)**. For the indoor experiment, samples were collected at 0, 2, 4, and 5 DAP. For the field experiment, samples were collected at 0, 14, 28, 40, 45, 61, and 72 DAP. T, tuber without bud eyes and shoots; BE/S, bud eyes plus shoots. Values are means ± SE (*n* = 3). Different letters indicate significant differences compared to 0 DAP for each tissue.

Since Suc is the main simple sugar present in Jerusalem artichoke T and BE/S (**Figures 4B,C**), we examined the activities of enzymes involved in Suc metabolism. VI enzyme activity increased, in T grown indoors, until 4 DAP and then decreased at 5 DAP while BE/S VI activity increased until 5 DAP (**Figure 5A**). NI activity displayed the opposite trend to VI activity in the indoor-grown T, reaching a minimum value at 4 DAP and increasing again at 5 DAP. The NI activity increased in BE/S up to 5 DAP. In field-grown Jerusalem artichokes (**Figure 5B**), the VI activity peaked at 45 DAP in T and at 72 DAP in BE/S. The NI activity gradually increased in T and BE/S (**Figure 5B**).

**FIGURE 6:**
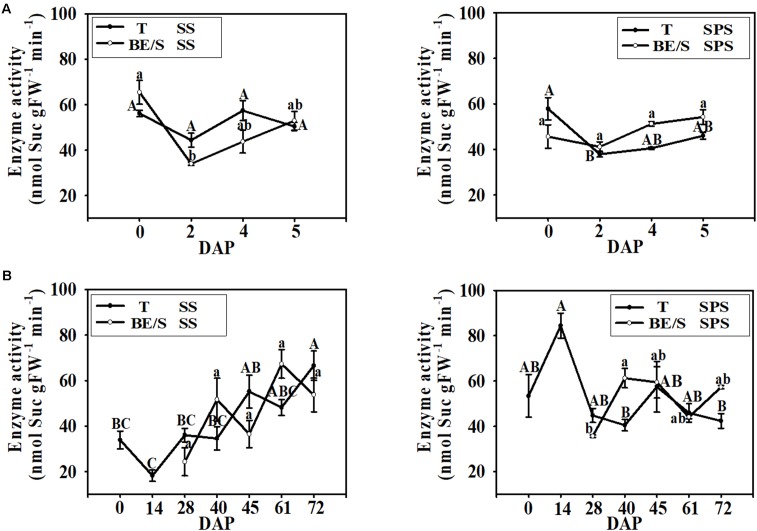
SS and SPS activities in T and BE/S grown indoors **(A)** and in the field **(B)**. For the indoor experiment, samples were collected at 0, 2, 4, and 5 DAP. For the field experiment, samples were collected at 0, 14, 28, 40, 45, 61, and 72 DAP. T, tuber without bud eyes and shoots; BE/S, bud eyes plus shoots. Values are means ± SE (*n* = 3). Different letters indicate significant differences compared to 0 DAP for each tissue.

In the indoor germination experiment, the SS activity in T remained relatively stable throughout germination (**Figure 6A**); however, the SS activity in BE/S was higher at 0 DAP than at the other time points examined. In contrast to SS activity, SPS activity was higher in T than in BE/S at the beginning of the experiment in the indoor-grown artichokes, and the activity in BE/S remained relatively stable until 5 DAP. In the field-grown artichokes, SS activity in T gradually increased throughout the experiment (**Figure 6B**). BE/S SS activity showed no marked differences between 28 and 61 DAP; however, T SPS activity was significantly lower at 40 DAP and 72 DAP as compared to 14 DAP. BE/S SPS activity increased until 40 DAP and remained stable later on.

## Discussion

Since most Jerusalem artichoke cultivars produce very few seeds with low viability, farmers mainly propagate this vegetable asexually via tubers. Time of germination correlates with external factors (such as temperature) and interior factors (such as enzyme activities or sugar content). Inulin is assumed to serve as a carbon source for growth before photosynthesizing leaves fully expand, but this was not investigated in detail so far. To clarify the situation, fructan metabolism was investigated in T and BE/S separately.

### Ht1-FEH II/III Function in Inulin Degradation During Tuber Germination

Even though the genes encoding the three *Ht1-FEH*s in Jerusalem artichoke have similar sequences, the different *Ht1-FEH*s have distinct spatio-temporal expression patterns and the three encoded enzymes have distinct reaction kinetics (Xu et al., 2015; Zhan et al., 2018). In our previous study, it was found that *Ht1-FEH I* transcripts were most abundant in above-ground parts throughout the growth period, while *Ht1-FEH II* transcripts accumulated in germinating tubers (Xu et al., 2015). In this study, *Ht1-FEH I* expression decreased during germination in both the indoor- and field-grown T, while *Ht1-FEH II* expression increased in T and BE/S. The recently identified Ht1-FEH III has a relatively high optimal temperature (55°C vs. 35°C for Ht1-FEH I and II). Unlike other enzymes in this family, it is able to hydrolyze Suc *in vitro* (Zhan et al., 2018). *Ht1-FEH III* expression generally increased in BE/S from indoor-grown artichokes during germination.

There are a number of observations and reasonings that argue against a model of local fructan synthesis in BE/S from tuber-imported Suc, followed by fructan degradation to sustain growth: (1) such synthesis and degradation would imply the loss of an excessive amount of ATP, as compared to direct import of fructans from the tubers; (2) typically, *de novo* fructan synthesis is always accompanied by temporal Glc accumulation (a 1-SST product) and this is not observed at any time point in BE/S; (3) the fructan profiles in BE/S are very similar to those of the T from which they originated, and (4) our calculations predict that the early 1-SST activities in BE/S are much too low to account for the total fructan levels observed in them. Taken together, this suggests that fructans in early BE/S are probably imported from T but this requires further investigation. Translocation of fructans through the phloem or xylem is currently under debate, since only a few reports point in this direction (Wang and Nobel, 1998; Sun et al., 2016).

The total Ht1-FEH activity changed only moderately in T and BE/S in indoor- and field-grown artichokes during germination, despite more drastic changes in *Ht1-FEHI-III* transcript levels (this study and a previous one: Xu et al., 2015) implying that different regulatory mechanisms are into play. A previous study (Marx et al., 1997) reported that the activities of Ht1-FEHs remained relatively stable from January to April, except for a decrease in February. The activities of Ht1-FEHs increased just after the tubers entered dormancy (from October to November) and during dormancy (from December to January) (Marx et al., 1997).

### Ht1-FFT and Ht1-SST Are Less Active in Germinating Tubers

The level of *Ht1-FFT* and *Ht1-SST* transcripts decreased or remained stable during germination in indoor- and field-grown tubers (**Figures 1**, **2**). In both of these growth environments, Ht1-FFT activity was higher in BE/S than in T. In indoor-grown artichokes, Ht1-SST activity was lower in BE/S than in T, but there were no prominent differences in Ht1-SST activity in field-grown T and BE/S, except at 40 and 61 DAP. In our previous study (Xu et al., 2015), it was found that Ht1-SST activity gradually decreased after germination, while Ht1-FFT activity remained stable in indoor-grown tubers. In a recent study, the activities of Ht1-FFT and Ht1-SST in Jerusalem artichoke tubers were reported to decrease during a 7-day germination period (Luo et al., 2018). These results confirm that Ht1-FFT and Ht1-SST do not have important roles during tuber germination.

### Lower DP Fructans Decreased During Germination in Field-Grown Artichokes, but Fru Only Increased at the End of Germination

During germination, the total soluble sugar content generally decreased in T and BE/S from indoor- and field-grown origin, most likely because these sugars are the energy source for growth. Additionally, the sugar profiles changed during tuber germination. During the short period of indoor germination, Fru and Glc contents did not change in T and BE/S, but the content of fructans with different DP (such as 1-K) gradually decreased (**Figures 4A–C**). In field-grown artichokes, the content of Glc and of different DP fructans followed the same trends as observed in the indoor-grown tubers, except that the Fru content sharply increased at 72 DAP (April). This is interesting, since the degradation product of fructan is Fru, but the Fru content did not change much during tuber germination. In agreement with our findings, a previous study found that the Fru content of tubers remained constant until April 1, but sharply increased by April 29, whereas the 1-FEH activity did not increase much in April (Marx et al., 1997). Furthermore, the relative content of DP 2–5 fructan first increased and then decreased during a 7-day germination period, but the content of higher DP fructans first decreased and then increased in Jerusalem artichoke tubers (Luo et al., 2018). The contents of Glc, Fru, and Suc were also found to decrease in potato tubers during germination (Dai et al., 2016).

### The Activities of Suc Metabolism Enzymes During Germination

Suc is the main simple sugar in T and BE/S of Jerusalem artichoke, suggesting that Suc plays an important role during germination (**Figure 5B**). Suc metabolism has been well documented (Winter and Huber, 2000; Koch, 2004; Ruan, 2014). A previous study reported that the Suc content in Jerusalem artichoke tubers decreased from January to April, and only increased during sprouting (Marx et al., 1997). Furthermore, we found that the Suc content was lower in BE/S than in T (**Figures 5A,B**). This argues against localized fructan synthesis, since 1-SST enzymes are essentially unsaturable, with Km’s in the range of 300–500 mM (Van den Ende et al., 1996). Accordingly, this fits well with a fructan/Suc translocation hypothesis. Possibly, Ht1-FEH III is involved in degradation of imported Suc and fructans in BE/S.

For comparison, it was previously shown that Suc and hexose contents were slightly increased in the sprouting buds and developing shoots of sugar beet (*Beta vulgaris*) after planting, but the activities of SS and INV increased (Verma et al., 2013).

### Buds Mobilize Incoming Sugars Faster Than Tubers

In our study, it was found that BE/S contained higher 1-FEH and 1-FFT activities than T, both in indoor- and field-grown artichokes (**Figure 3**). 1-FFT also functions in fructan degradation, since this enzyme can transfer fructosyl groups from high DP fructans to low DP fructans and Suc. It was previously reported that fructan profiles changed during Jerusalem artichoke tuber sprouting and that fructosyl moieties present in high DP fructans (>5) may be transferred to DP 2–5 fructan (Luo et al., 2018). In our study, fructan content (1-K, N, and 1^F^-N) in BE/S was generally lower than in T (**Figures 4B,C**), indicating that BE/S degraded inulin faster. Interestingly, the content of Fru did not substantially increase until the end of germination, suggesting that the rate of inulin degradation in T and BE/S is matched to sugar demands to sustain growth processes (shoot elongation).

## Conclusion

In this study, we characterized the transcription of genes involved in sugar metabolism and the activity of the encoded enzymes, as well as the sugar content, during Jerusalem artichoke tuber germination. The expression of genes involved in inulin metabolism, including *Ht1-FEH*, *Ht1-SST*, and *Ht1-FFT* was rapidly altered during germination, while the activities of most of the enzymes examined (1-FEH, 1-FFT, and INV) remained relatively stable in shoots and tubers. While the inulin content slowly decreased, the Fru content only increased at the end of germination. The activities of 1-FEH and 1-FFT were higher in BE/S than in T, suggesting that BE/S degrade imported fructans from tubers more efficiently to sustain shoot growth. Ht1-FEH III may be a key player in Suc and fructan degradation in BE/S. As the sugar dynamics of Jerusalem artichoke tubers germinated indoors are similar to those germinated in the field, future germination studies can be conducted indoors, which would simplify the procedures and make them more controllable.

## Author Contributions

ML and WVdE conceived and designed the experiments, and wrote the manuscript. JJ, JW, MZ, XR, and WZ performed the experiments. ZS and HZ analyzed the data. YY contributed reagents, materials, and analysis tools.

## Conflict of Interest Statement

The authors declare that the research was conducted in the absence of any commercial or financial relationships that could be construed as a potential conflict of interest.

## Supplementary Material

FIGURE S1 Photographs of Jerusalem artichokes grown indoors **(A)** and in the field **(B)**. For the indoor experiment, samples were collected at 0, 2, 4, and 5 DAP. For the field experiment, samples were collected at 0, 14, 28, 40, 45, 61 and 72 DAP. Horizontal bar represents 1 cm.

FIGURE S2 Fructan profiles in T **(A)** and BE/S **(B)** in Jerusalem artichokes grown indoors (5 DAP). DAP: days after planting. F, fructose; G, glucose; S, sucrose; 1-K, 1-kestose; N 1, 1-nystose; 1^F^-N, 1^F^-fructofuranosylnystose.

FIGURE S3 Fructan profiles in T **(A)** and BE/S **(B)** in Jerusalem artichokes grown in the field (72 DAP). DAP, days after planting; F, fructose; G, glucose; S, sucrose; 1-K, 1-kestose; N 1, 1-nystose; 1^F^-N, 1^F^-fructofuranosylnystose.

TABLE S1 Average temperature in Nanjing from January to April in 2017. (source: http://tianqi.eastday.com/).

TABLE S2 Primers used for qPCR analysis.

## Abbreviations

1-FFT: Fructan:fructan 1-fructosyltransferase
1^F^-N: 1^F^-fructofuranosylnystose
1-K: 1-kestose
1-SST: Sucrose:sucrose 1-fructosyltransferase
BE/S: Bud eyes plus shoots
DAP: Days after planting
DP: Degree of polymerization
FEH: Fructan exohydrolase
Fru: Fructose
Glc: Glucose
INV: Invertase
N 1: 1-nystose
NI: Alkaline/Neutral invertase
SPP: Sucrose 6-phosphate phosphatase
SPS: Sucrose 6-phosphate synthase
SS: Sucrose synthase
Suc: Sucrose
T: Tuber without BE/S
VI: Vacuolar invertase
