# Supplementary material for: Characterization of Fructan Metabolism During Jerusalem Artichoke (Helianthus tuberosus L.) Germination
Source: Front Plant Sci. 2018 Sep 19;9:1384. doi: 10.3389/fpls.2018.01384 (PMC6156445; doi:10.3389/fpls.2018.01384)
Supplement: FIGURE S1 — Photographs of Jerusalem artichokes grown indoors (A) and in the field (B). For the indoor experiment, samples were collected at 0, 2, 4, and 5 DAP. For the field experiment, samples were collected at 0, 14, 28, 40, 45, 61 and 72 DAP. Horizontal bar represents 1 cm. [file Data_Sheet_1.PDF]

Supplementary Table 1. Average temperature in Nanjing from January to April in 2017. (source: http://tianqi.eastday.com/).

Month	Average maximum temperature (°C)	Average minimum temperature (°C)
January	10 ± 3.31	2 ± 2.56
February	12 ± 4.26	2 ± 3.04
March	15 ± 3.00	6 ± 2.75
April	19 ± 3.08	11 ± 2.81

Supplementary Table 2. Primers used for qPCR analysis.

Gene	Primer	Primer sequence (5'-3')
Ht1-FEH I	qPCR Ht1-FEH I-F	ACCGATCAGTTTCTCCTTGCTACC
	qPCR Ht1-FEH I-R	CCATGCCCATAACACCCTTC
Ht1-FEH II	qPCR Ht1-FEH II-F	ACCCAATAATAACCCCACCCG
	qPCR Ht1-FEH II-R	CGTACCGAGTCCAGGTTACAA
Ht1-FEHIII	qPCR Ht1-FEH III-F	AATGGTGAACCCGTAATC
	qPCR Ht1-FEH III-R	CCCACTCACTTAGTAGCG
Ht1-FFT	qPCR Ht1-FFT-F	ACCCGCTTCTTATTGAGT
	qPCR Ht1-FFT-R	TGTATTGCCACGTTTAGTT
Ht1-SST	qPCR Ht1-SST-F	GTAAGCGTAGGGAAATGG
	qPCR Ht1-SST-R	CTTCGCAGCGTTGTAGAT
Ht Actin	qPCR Ht Actin-F	ATGTATGTAGCCATCCAGG
	qPCR Ht Actin-R	TGTTAGGTCACGCCCAG

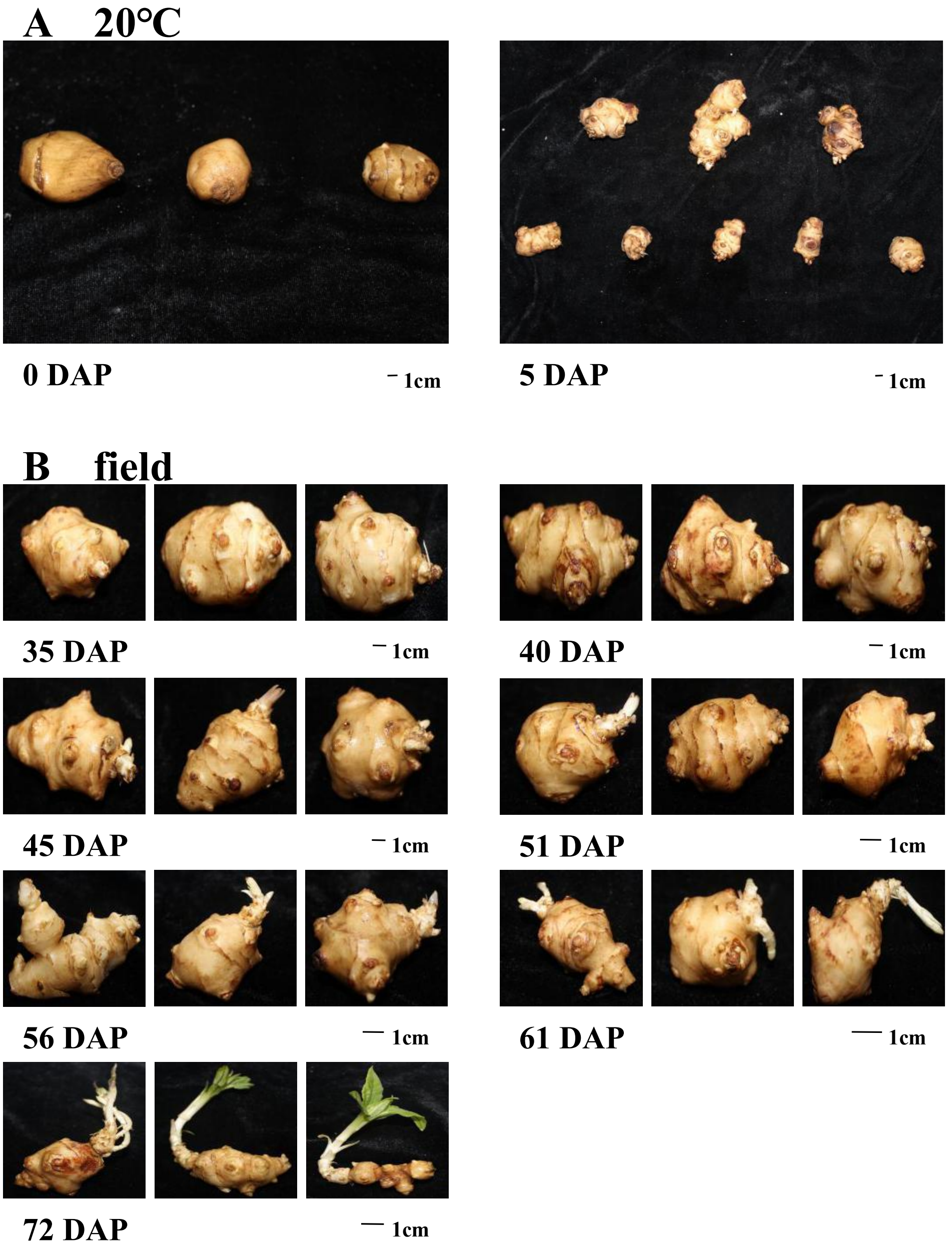

Supplementary Fig. 1.

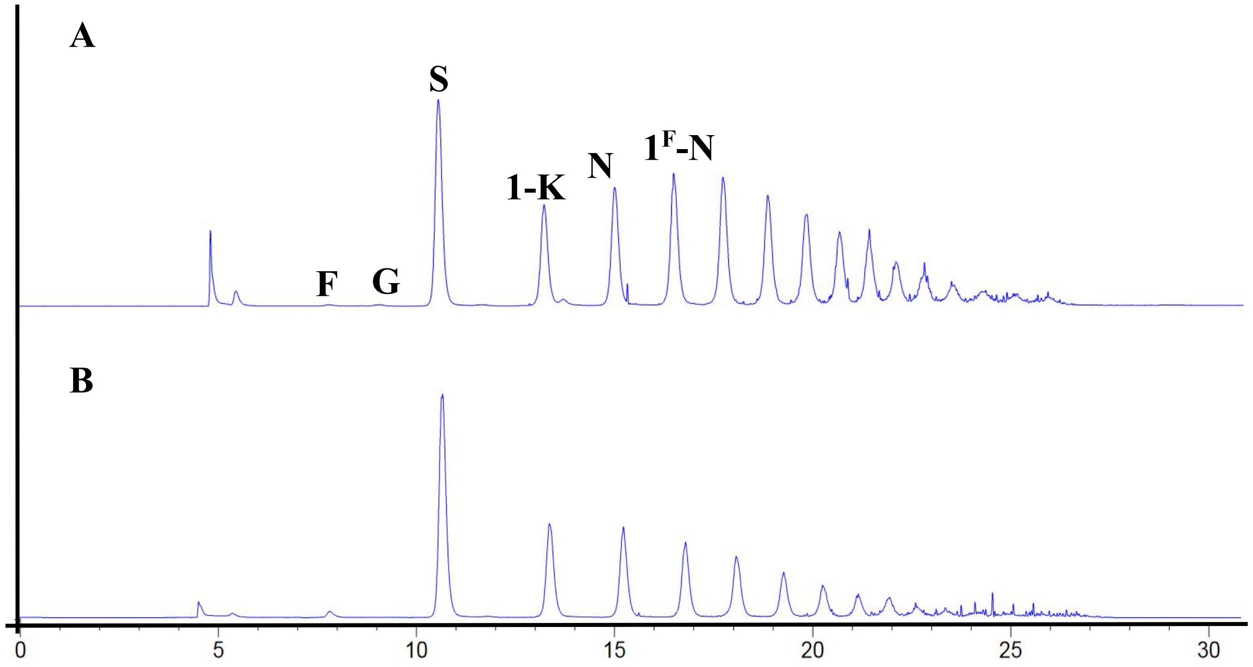

Supplementary Fig. 2.

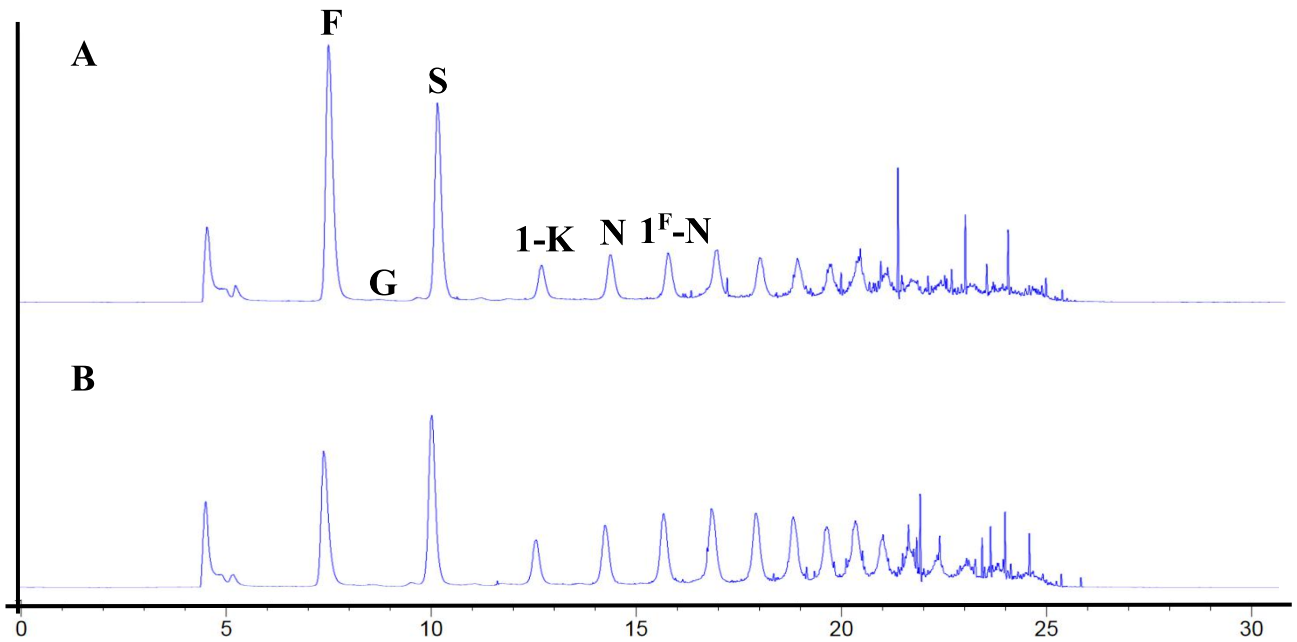

Supplementary Fig. 3.
